# Only two in five pregnant women have adequate dietary diversity during antenatal care at Hiwot Fana Specialized University Hospital in Eastern Ethiopia

**DOI:** 10.1017/jns.2024.7

**Published:** 2024-03-21

**Authors:** Sinetibeb Mesfin, Dawit Abebe, Hirut Dinku Jiru, Seboka Abebe Sori

**Affiliations:** 1 School of Nursing and Midwifery, College of Health and Medical Sciences, Haramaya University, Harar, Ethiopia; 2 School of Nursing and Midwifery, College of Health and Medical Sciences, Jigjiga University, Jigjiga, Ethiopia; 3 Department of Midwifery, College of Medicine and Health Sciences, Wolkite University, Wolkite, Ethiopia

**Keywords:** Awareness of dietary diversity, Dietary diversity, Eastern Ethiopia, Pregnant women

## Abstract

Dietary diversification is a sustainable and appealing strategy for pregnant women to ensure a balanced dietary intake. In Ethiopia, despite the implementation of various nutritional initiatives, inadequate dietary diversity remains a significant factor contributing to adverse birth outcomes. Thus, this study aimed to assess the dietary diversity and associated characteristics among pregnant women attending antenatal care in Eastern Ethiopia. Institution-based cross-sectional study was conducted from April 28 to May 28, 2021. A total of 420 pregnant women were selected using a systematic random sampling technique. We used the adjusted odds ratio (AOR) and a 95% confidence interval to estimate the strength of the association. We used a *p*-value of 0.05 to declare statistical significance. Only 35.0% (95% CI: 30.5, 39.5) of the 420 pregnant women involved in this study received appropriate dietary diversity. Having an educational level of college and above (AOR 3.01, 95% CI: 1.19–7.5), being an urban dweller (AOR = 3.57, 95% CI: 1.68–7.52), eating three meals and above (AOR = 7.62, 95% CI: 2.88–9.03), and having ≤4 family sizes (AOR = 9.33, 95% CI: 4.06–10.4) were significantly associated with an adequate dietary diversity score among pregnant women. This study found that pregnant women had inadequate overall consumption of a diversified diet. Increasing meal frequency, enhancing women’s education, raising awareness of dietary diversity among rural inhabitants, and offering counselling on family planning utilisation during ANC services are all beneficial in promoting dietary diversity among pregnant women.

## Introduction

Dietary diversity is consuming different food groups within 24 h of the assessment.^(1)^ It illustrates the impression that increasing food variety and quality helps people get enough of the essential nutrients they need.^(2)^ Pregnancy adds to a woman’s normal nutritional needs by increasing nutrient requirements to maintain maternal metabolism demand and foetal growth.^(3)^ Proper nutrition before and during pregnancy plays a crucial role in decreasing adverse outcomes like intrauterine growth restriction, low birth weight, preterm delivery, intrauterine foetal death, and congenital anomalies.^(4)^ A monotonous diet pattern increases the likelihood of poor micronutrient intake, whose deficiency can result in maternal undernutrition.^(5)^ In addition, pregnant women often have low and decreasing levels of micronutrients in their blood. To address this issue, it is advisable to promote the maintenance of dietary diversity as a beneficial and potentially sustainable strategy for achieving a well-rounded nutrient intake during pregnancy.^(6)^ Dietary diversity is therefore considered very crucial to providing an adequate supply of nutrients for both the mother and the developing foetus.^(7,8)^

Maternal nutritional status during pregnancy has a lasting, multigenerational impact that affects not only the health and survival of the women but also that of their children.^(9)^ A pregnant woman with higher dietary diversity assures micronutrient adequacy that minimises the risk of developing a deficiency or excess of any one nutrient. Consuming micronutrient-rich foods such as fruits, vegetables, meat, and fortified foods throughout pregnancy is critical for a healthy pregnancy and improved maternal health.^(10)^

According to the WHO report, the burden of micronutrient malnutrition among pregnant women is still alarmingly high across regions and countries.^(4)^ Micronutrient deficiencies are estimated to account for about 7.3% of the global burden of diseases, and millions of pregnant women are deficient in vitamin A, iron, folate, zinc, or iodine.^(5)^

In Africa, where maternal mortality is exponentially higher than in other regions, the extent and consequence of micronutrient deficiency among pregnant women remain unacceptably high.^(9)^ In sub-Saharan Africa, maternal micronutrient deficiencies are persistent and a key contributor to morbidity, mortality, and poor birth outcomes such as preterm birth, stillbirth, and low birth weight.^(11)^

In a systematic review, Azene (2021) found that only 41% of pregnant women in Ethiopia had adequate dietary diversity.^(11)^ Despite the implementation of different nutritional policies, micronutrient deficiencies continued to account for a substantial proportion of poor birth outcomes in Ethiopia.^(12,13)^ Even though some studies were conducted in some parts of Ethiopia, the prevalence of adequate dietary diversity and determinant factors varies across studies. Thus, a better understanding of the factors associated with dietary diversity is critical for preventing the risk of micronutrient deficiencies and helps design appropriate interventions by health policymakers to tackle the problem. Therefore, this study aimed to assess the dietary diversity and associated factors among pregnant women attending antenatal care at Hiwot Fana Specialized University Hospital, Eastern Ethiopia.

## Materials and methods

### Study setting, design, and period

An institution-based cross-sectional study was conducted at Hiwot Fana Specialized University Hospital (HFSUH), which is located in Harar, 526 kilometres to the east of Addis Ababa. HFSUH is a major referral hospital for the Eastern part of Ethiopia, including Dire Dawa City Administration, Oromia, Ethiopian Somali, and Harari regions. The hospital has 312 beds and 15 case teams to provide services in all specialties. We conducted the study from April 28, 2021, to May 28, 2021.

### Source population, study population, and eligibility criteria

All pregnant women receiving antenatal care (ANC) at Hiwot Fana Specialized University Hospital were the source population. All pregnant women receiving ANC at Hiwot Fana Specialized University Hospital during the data collection period were the study population. Pregnant women selected by systematic random sampling technique and participated in the actual data collection interview were the study units. We included all pregnant women receiving ANC at Hiwot Fana Specialized University Hospital during the data collection period. However, we excluded those who were unable to provide information due to a serious illness from the study.

### Sample size determination and sampling procedure

A single population proportion formula was used to calculate a sample size, assuming a 95% confidence level, 5% margin of error, 10% non-response rate, and 53% proportion of adequate dietary diversity from a study conducted in Gojjam, Ethiopia.^(14)^ Accordingly, the total sample size was 420. The study participants were selected using a systematic random sampling technique. The sampling interval K was determined by dividing the average number of pregnant women who attended antenatal care follow-up per month at HFSUH by the desired sample size, (i.e. k^th^ value 1166/420 = 3). After randomly selecting the first study participant, subsequent study participants were chosen at every third interval until the total sample size was attained and interviewed at exit from ANC service.

### Measurement and data collection tools

Data were obtained from face-to-face interviews using a structured questionnaire. The questionnaire was adapted from the Food and Agriculture Organization (FAO) following guidelines outlined for the calculation of minimum dietary diversity for women of reproductive age, Food and Nutrition Technical Assistance (FANTA) Project household food insecurity access scale,^(1,15)^ and from different literature, which was pertinent to the topic.^(12–14)^ It consisted of three groups of participants’ characteristics, namely, socio-demographic, dietary information, and food security information, which was prepared in English and translated to local languages (Afan Oromo and Amharic) before data collection. We evaluated respondents’ dietary diversity by relying on the pregnant mother’s recall of food items consumed in the 24 h preceding the survey. A total of ten groups (including (a) grains, white roots and tubers, and plantains (also known as starchy staples); (b) pulses (beans, peas, and lentils); (c) nuts and seeds; (d) dairy; (e) meat, poultry, and fish; (f) eggs; (g) dark green leafy vegetables; (h) other vitamin A-rich fruits and vegetables; (i) other fruits; and (j) other vegetables) were used. Each group was assigned a score of 1 point if they consumed any of the foods in each subgroup at least once in the past 24 h and 0 points if they did not consume the food at all. The minimum dietary diversity score was calculated by adding the number of food categories consumed for 24 h. Participants who consumed five food groups were considered to have adequate dietary diversity, whereas those who consumed less than five food groups were considered to have inadequate dietary diversity.

### Quality control

We provided 3 days of training for data collectors on the questionnaire, how to interview, and sampling procedures. A pretest was done on 5% of the samples at Dilchora Hospital with some time gap to decrease information contamination before the actual data collection, and an amendment was made accordingly. A total of six BSc midwives were recruited for the data collection. The principal investigators closely supervised the data collection process.

### Data process and analysis

We coded, cleaned, and entered the collected data into EpiData version 3.1 and subsequently exported it to SPSS version 22 (IBM SPSS Statistics, 2013) for analysis. The descriptive analysis was done using frequency tables. Employing binary logistic regression, we used bivariate analysis to establish the relationship between each independent variable and the outcome variable. We considered all variables with a *p*-value of 0.25 in bivariate analysis eligible for multivariable logistic regression analysis to control for potential confounders and identify true predictors of dietary diversity. We used the variance inflation factor (VIF) and tolerance to test for multicollinearity and found no indication of collinearity effects. We used the Hosmer–Lemeshow goodness-of-fit test to assess model adequacy, and the test yielded a negligible result (*p* = 0.677), indicating that the model was fit. Finally, we used an adjusted odds ratio and a 95% confidence interval to determine the degree of association between the outcome variable and the independent variables. We used a *p*-value of 0.05 to declare statistical significance.

### Ethical approval

We conducted this study following the guidelines outlined in the Declaration of Helsinki. The Institutional Health Research Ethics Review Committee of Haramaya University, College of Health and Medical Sciences (HU-IHRERC), approved the technical proposal for this study (Ref/No: COH.M.S/100/12940/21). We obtained a permission letter from the hospital administrator. We provided participants with an explanation of the purpose of the research. We told the respondents their participation was fully voluntary and they could opt out at any time or stage of the interview. All study participants provided written informed consent to participate in the study.

## Results

### Socio-demographic and economic characteristics

In this study, a total of 420 participants were involved with a 100% response rate. The mean (±SD) age of participants was 29.6(±7.1) years. Almost all of the participants 407 (96.9%) were married, and half (52.4%) of them were Oromo. Only 79 (18.8%) of respondents couldn’t read or write, and over two-thirds (74.1%) of respondents were Muslim. In terms of occupation, 128 (30.5%) of respondents were housewives, and 87 (20.7%) had less than 2000 Ethiopian birr (37.95 USD) for monthly family member expenditures. Three hundred sixty-seven (87.4%) of the respondents had families of less than four members, two-thirds (74%) lived in cities, and half (56.8%) of the household heads were between the ages of 30 and 39.

The vast majority of 382 (91 %) respondents’ food sources were found to be purchased from the market. The majority of study participants, 353 (84%), sourced their water from taps, followed by 60 (14.3%) and 7 (1.7%) study participants who get it via pumps and protected wells, respectively. Among the study participants, 293 (69.8%) of them had a private latrine facility, and 141 (48.1%) of them had pit latrines with a slab. About 246 (58.6%) of the study participants engaged in home gardening, and 358 (86.1%) of the respondents had no comorbid illnesses before the data collection period of 4 weeks (Table 1).

**Table 1.** Socio-demographic characteristics of pregnant women attending antenatal care at Hiwot Fana Specialized University Hospital, Harar, Eastern Ethiopia, 2021

Variables	Categories	Frequency	Percentage
Age of mother	≤29 years	187	44.5
	30–39 years	207	49.3
	≥40 years	26	6.2
Marital status	Married	407	96.9
	Single	7	1.7
	Divorced/widowed	6	1.4
Religion	Orthodox	151	36
	Muslim	236	56.2
	Protestant	30	7.1
	Other*	420	0.7
Educational status of the mother	No formal education	79	18.8
	Read and write	104	24.8
	Primary education	62	14.8
	Secondary education	93	22.1
	College and above	82	19.5
Occupation of the mother	Housewife	128	30.5
	Governmental employee	122	29
	Daily labour/farmer	105	3.8
	NGO	16	25
	Self-employee	49	11.7
Parity	≤2	285	67.9
	>2	135	32.1
Age of the household’s head	≤29 years	79	19.2
	30–9 years	234	56.8
	≥40 years	99	24
Household income in Ethiopian birr (ETB)	<2000	87	20.7
	2000–3500	132	31.4
	>3500	201	47.9
Residence	Urban	311	74
	Rural	109	26
Family size	≤4	53	12.6
	>4	367	87.4
Source of water	Tap water	353	84
	Pumping water	60	14.3
	Protected well	7	1.7
Latrine	Yes	293	69.8
	No	127	30.2
Types of latrine	Ventilated, improved pit latrine	34	11.6
	Pit latrine with slab	141	48.1
	Pit latrine without a slab	76	25.9
	Open pit	42	14.3
Practicing home garden	Yes	246	58.6
	No	174	41.4
Sources of food	Market	382	91
	Farm/garden	35	8.3
	NGO	2	.4
	Relative or friend	1	.2
Illness in last 4 weeks	Yes	54	13.1
	No	358	86.9
Type of illness	Malaria	22	31.5
	HIV	17	40.7
	Other**	15	27.8

HIV, human immunodeficiency virus; NGO, non-governmental organization; 1ETB, 0.018 USD.

### Consumption of food groups, food security, and comorbidity status

In terms of the food groups consumed by pregnant women in the preceding 24 h, the majority of the women 378 (90%) consumed starchy staples. Of them, 110 (26.2%) consumed vitamin A-rich fruit and vegetables, and 154 (36.7%) consumed nuts and seeds. Moreover, foods of animal products were highly consumed by the study participants, with two-thirds (74.8%) consuming meat, poultry, and fish, half (54.3%) consuming eggs, and 237 (56.4%) consuming dairy products. About 147 (35%) of the study population consumed dark green leafy vegetables, and 110 (26.2%) consumed other vegetables (onion, tomato, and eggplants). About 264 (62.9%) consumed pulses, while only 70 (16.7%) consumed fruit. One hundred fifty-three (36.4%) of study participants had secured food, and half (58.6%) of respondents consumed two meals and ate between meals per day (Table 2).

**Table 2.** Consumption of pregnant women attending antenatal care at Hiwot Fana Specialized University Hospital, Eastern Ethiopia, 2021

Food groups		Frequency	Percentage
Starchy staples	Yes	378	90
	No	42	10
Vitamin A-rich fruit and vegetables	Yes	302	71.9
	No	118	28.1
Other vegetables	Yes	110	26.2
	No	310	73.8
Dark green leafy vegetables	Yes	147	35
	No	273	65
Fruit	Yes	70	16.7
	No	350	83.3
Meat, poultry, fish	Yes	314	74.8
	No	106	25.2
Eggs	Yes	228	54.3
	No	192	45.7
Pulses	Yes	264	62.9
	No	156	37.1
Dairy	Yes	237	56.4
	No	183	43.6
Nuts and seeds	Yes	154	36.7
	No	266	63.3
Dietary diversity	Adequate dietary diversity	147	35.0
	Inadequate dietary diversity	273	65.0
Food security status	Food secured	153	36.4
	Food unsecured without hunger food	221	52.6
	Unsecured with hunger	46	11.0
Food eating pattern	Three meals and above	160	38.1
	Two meals and eating between-meal	246	58.6
	Two meals only or below	14	3.3

### Prevalence of dietary diversity score based on 24-hour recall

**Fig. 1.**
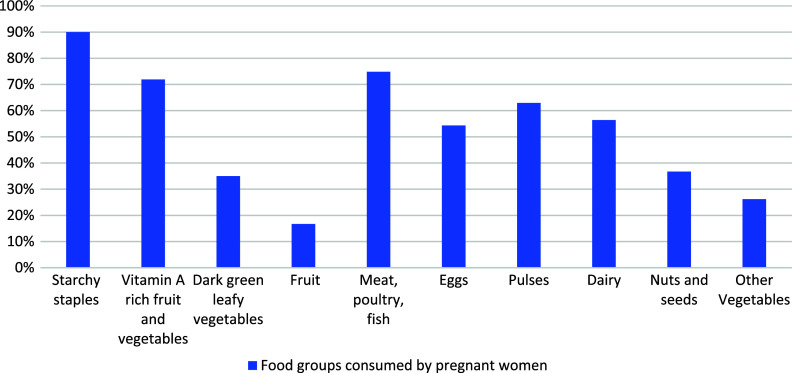
Food groups consumed by pregnant women attending antenatal care at Hiwot Fana Specialized University Hospital, Harar, Eastern Ethiopia, 2021.

In the current study, the mean dietary diversity (±SD) score of pregnant mothers was 3.71 ± 2.57 SD with scores ranging from 3 to 8 food groups. The overall prevalence of adequate dietary diversity practice among pregnant women was found to be 35 % (95% CI 30.5 – 39.5) (Fig. 1).

### Factors associated with dietary diversity

We used both bivariate and multivariate binary logistic regressions to investigate the relationship between dependent and independent variables. During the bivariate analysis, the educational level, occupation, age, family size, access to latrine, residency, and eating pattern of women were identified as significantly correlated with the dietary diversity of pregnant women in the study area. The multivariable analysis demonstrated that educational level, family size, residency, and eating behaviour were significantly associated with adequate dietary diversity after controlling for potential confounders (Table 3).

**Table 3.** Factors associated among pregnant women attending antenatal care at Hiwot Fana Specialized University Hospital, Eastern Ethiopia, 2021

Variables	Category	Dietary Diversity		COR (95%CI)	AOR (95%CI)
		Adequate	Inadequate		
Residence	Urban	117	194	1.58(0.98,2.56)	3.57(1.68,7.62)*
	Rural	30	79	1.00	1.00
Latrine	Yes	95	198	0.69(0.45,1.06)	1.06(0.603,1.87)
	No	52	75	1.00	1.00
Family size	≤4	40	13	7.48(3.85,14.54)	9.33(4.06,10.4)**
	>4	107	260	1.00	1.00
Eating pattern	Two meals or below	4	10	1.00	1.00
	Two meals and between-meal	43	203	0.53(0.16,1.77)	0.612(0.16,2.37)
	Three meals and above	100	60	4.16(1.25,13.87)	7.62(2.88,9.03)**
Women age	<29	91	96	1.79(0.76,4.22)	0.33(0.105,1.06)
	30–39	47	160	0.56(0.23,1.33)	0.35(0.12,1.008)
	>40	9	17	1.00	1.00
Occupation of wife	Housewife	46	82	1.00	1.00
	Civil servant	44	78	1.009(0.6,1.7)	0.81(0.41,1.6)
	NGO employee	4	12	0.59(0.18,1.95)	0.33(0.81,1.33)
	Farmer/daily labour	43	62	1.24(0.73,2.1)	1.77(0.83,3.76)
	Self-employee	10	39	0.46(0.21,1.1)	0.54(0.18,1.23)
Women educational level	No formal education	23	56	1.00	1.00
	Read & write	26	78	.812(0.42,1.57)	1.36(0.59,3.14)
	Primary education	22	40	1.34(0.66,2.73)	1.05(0.42,2.61)
	Secondary education	33	60	1.34(0.703,2.55)	1.81(0.75,4.39)
	College and above	43	39	2.68(1.4,5.15)	3.016(1.19,7.6)*

* *P-value <0.05*.

** *P-value* = *0.000*; CI, confidence interval; COR, crude odds ratio; AOR, adjusted odds ratio.

Participants with a college education or above were three times more likely to have adequate dietary diversity in their diet compared to participants whose education status was elementary school (AOR = 3.01, 95% CI: 1.19 – 7.6). Those who had three meals and above were seven times more likely to have adequate dietary diversity in their diet compared with those who had two meals only and below (AOR = 7.62, 95% CI: 2.88 – 9.03). Respondents who live in urban areas were 3.57 (AOR = 3.57, 95% CI:1.68 – 7.62) times more likely to have adequate dietary diversity in their diet than their counterparts. Those pregnant women who have ≤4 family sizes were nine times more likely (AOR = 9.33, 95% CI: 4.06 – 10.4) to have adequate dietary diversity than those who have >4 (Table 3).

## Discussion

The study revealed that about 35% of the pregnant women had consumed ≥5 food groups (adequate dietary diversity), whereas 65% had consumed <5 food groups (inadequate dietary diversity) in the last 24 h. The prevalence of dietary diversity in this study is slightly higher than findings from studies conducted in Shashemane, Ethiopia (25.4%),^(16)^ and Kenya (20%).^(7)^ This discrepancy might be due to variations in the study period, geographical area, socioeconomic, and reporting (hence self-reporting). But it is lower than studies conducted in Gurage zone (42.1%),^(17)^ Hossana (42.6%),^(18)^ Bale (44.8%),^(19)^ Gojjam (55%),^(14)^ Alamata (61.2%),^(20)^ and Ghana (46 %).^(21)^ The discrepancy may be caused by variations in how dietary diversity was measured and categorised, eating habits, and other socio-demographic factors of women that are found in various areas of the country. Furthermore, socioeconomic status, seasonality, and geographic location may all have a role.^(21)^

The study findings indicated that pregnant women with college or higher education had adequate dietary diversity in their diets, compared to those without formal education. This aligns with similar observations in Alamat, Eastern Gojjam,^(20)^ and Hossana, Ethiopia.^(18)^ This might be because educated women are more likely to comprehend nutritional information and incorporate a range of food categories in their meals to achieve the desired level of dietary diversity. Additionally, educated women may participate in income-generating activities, allowing them to buy a variety of foods and adopt a healthy eating attitude.^(22)^

In our study, respondents who consumed three meals and above had adequate dietary diversity in their diet compared with those who had two meals only and below, which is in line with the findings of studies done in Alamata and Hadiya, Ethiopia.^(18,20)^ This might be because increasing the frequency of meals taken by women can help them consume a variety of foods that promote dietary diversity.^(15)^ Another reason might be the impact of their economic status on diet diversification. Women with a better socioeconomic status may be able to purchase a wider range of food categories more easily, allowing them to increase meal frequency while also improving their nutritional patterns.^(23)^

Similar to a study in Bale, Ethiopia,^(19)^ living in an urban environment is strongly associated with adequate dietary diversity within their diet when compared to individuals who live in a rural area. This could be because city dwellers have easier access to media outlets that disseminate health information and promote public awareness of dietary diversity. Another factor could be that people who live in cities have easy access to well-established marketplaces where they can easily purchase a variety of food items.^(24)^

Pregnant women who have ≤4 family sizes had high dietary diversity in their diet compared with those who have >4 family sizes, and this is consistent with data from Bale, Ethiopia.^(19)^ This might be because families with fewer members will have a higher chance of having physical and financial access to sufficient food that is safe and nutritious to meet their dietary demands.^(4)^ Furthermore, large family size has an impact on intra-household food distribution, which may necessitate limiting the type and quantity of food groups.^(25)^

### Limitations of the study

The study acknowledges a limitation in assessing dietary diversity, as it relied on participants’ recall. Additionally, the fluctuation of food availability in households across seasons may impact the measured dietary diversity. So, recall bias could not be ruled out completely, and the 24-hour dietary recall may not truly represent the usual intake. Since the study was conducted within the institution, generalising to the entire population is difficult. The MDD-W indicator used in this study was originally designed for women of reproductive age, and currently, no specific MDD indicator exists for pregnant women. Therefore, it is unknown whether they work for pregnant women or not.

### Conclusion

**Fig. 2.**
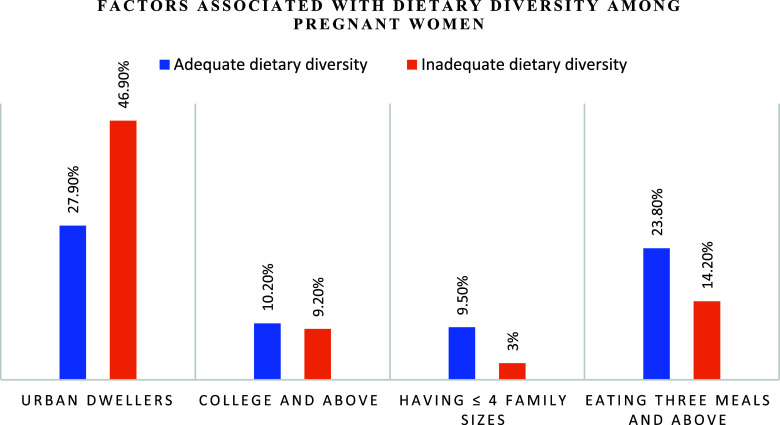
Factors associated with dietary diversity among pregnant women attending antenatal care at Hiwot Fana Specialized University Hospital, Eastern Ethiopia, 2021.

This study found that only two in five pregnant women have adequate dietary diversity during antenatal care at Hiwot Fana Specialized University Hospital in Eastern Ethiopia. Factors such as the mother’s level of education, family size, place of residence, and eating habits all had significant effects on dietary diversity among pregnant women. Therefore, promoting women’s education, raising awareness of dietary diversity among rural residents, increasing meal frequency, and providing counselling on family planning utilisation during ANC services are all beneficial in promoting dietary diversity among pregnant women (Fig. 2).

## Abbreviations

**AOR:** Adjusted odds ratio; **ANC:** Antenatal care; **CI:** Confidence interval; **COR:** Crude odds ratio; **FANTA:** Food and Nutrition Technical Assistance Project; **FAO:** Food and Agriculture Organization; **HFSUH:** Hiwot Fana Specialized University Hospital; **MDD:** Minimum dietary diversity; **WHO:** World Health Organization

## References

[1] FAO & FHI360. Minimum Diet Diversity for Women: A Guide for Measurement. Rome: Food and Agriculture Organization; 2016.

[2] Madzorera I , Isanaka S , Wang M , et al. Maternal dietary diversity and dietary quality scores in relation to adverse birth outcomes in Tanzanian women. Am J Clin Nutr. 2020;112(3):695–706. 32651998 10.1093/ajcn/nqaa172 PMC7458779

[3] Diana R , Christianti DF , Anwar F , et al. Food suggestions, meal frequency, and dietary diversity among pregnant women: a quantitative study. Future Food: J Food, Agric Soc. 2020;8(3):1–12.

[4] WHO. WHO | Micronutrients [Internet]. WHO. [cited 2021 JULY 16], 94(6). https://www.who.int/health-topics/micronutrients#tab=tab_1

[5] Ayensu J , Annan R , Lutterodt H , et al. Prevalence of anemia and low intake of dietary nutrients in pregnant women living in rural and urban areas in the Ashanti region of Ghana. PloS One. 2020;15(1):e0226026. 31978048 10.1371/journal.pone.0226026 PMC6980408

[6] World Health Organization (WHO). WHO Recommendations on Antenatal Care for a Positive Pregnancy Experience: Summary. Geneva, Switzerland: World Health Organization (WHO); 2018. Licence: CC BY-NC-SA 3.0 IGO.

[7] Willy K , Judith K , Peter C . Dietary diversity, nutrient intake, and nutritional status among pregnant women in Laikipia County, Kenya. Int J Health Sci Res. 2016;6(4):378–385.

[8] Custodio E , Kayikatire F , Fortin S , et al. Minimum dietary diversity among women of reproductive age in urban Burkina Faso. Matern Child Nutr. 2020;16(2):e12897. 31856424 10.1111/mcn.12897 PMC7083435

[9] Darnton-Hill I . Global Burden and Significance of Multiple Micronutrient Deficiencies in Pregnancy. Nestle Nutr Inst Workshop Ser. 2012: 49–60. 10.1159/000337421 25825295

[10] Tefera W , Brhanie TW , Dereje M. Dietary Diversity Practice and Associated Factors Among Pregnant Women Attending ANC in Kolfe Keranyo Sub-City Health Centers. Addis Ababa: Ethiopia. medRxiv; 2020.

[11] Azene AG , Aragaw AM , Wubetie HT , et al. Dietary diversity among pregnant women and associated factors in Ethiopia: systematic review and meta-analysis. PloS One. 2021;16(6):e0251906. 34111140 10.1371/journal.pone.0251906 PMC8191951

[12] Zerfu TA , Biadgilign S. Pregnant mothers have limited knowledge and poor dietary diversity practices, but the favorable attitude towards nutritional recommendations in rural Ethiopia: evidence from a community-based study. BMC Nutr. 2018;4(1):1–9. 32153904 10.1186/s40795-018-0251-x PMC7050941

[13] Zerfu TA , Umeta M , Baye K. Dietary diversity during pregnancy is associated with reduced risk of maternal anemia, preterm delivery, and low birth weight in a prospective cohort study in rural Ethiopia. Am J Clin Nutr. 2016;103(6):1482–1488. 27169832 10.3945/ajcn.115.116798

[14] Yeneabat T , Adugna H , Asmamaw T , et al. Maternal dietary diversity and micronutrient adequacy during pregnancy and related factors in East Gojjam Zone, Northwest Ethiopia, 2016. BMC Pregnancy Childbirth. 2019;19(1):1–9. 31092223 10.1186/s12884-019-2299-2 PMC6521398

[15] Kearney J. Food consumption trends and drivers. Philos Trans R Soc B: Biol Sci. 2010;365(1554):2793–2807. 10.1098/rstb.2010.0149 PMC2935122 20713385

[16] Desta M , Akibu M , Tadese M , et al. Dietary diversity and associated factors among pregnant women attending antenatal clinic in Shashemane, Oromia, Central Ethiopia: a cross-sectional study. J Nutr Metab. 2019;2019:1–7. 10.1155/2019/3916864 PMC6434279 30993019

[17] Gudeta TG , Terefe AB , Mengistu GT , et al. Determinants of dietary diversity practice among pregnant women in the Gurage Zone, Southern Ethiopia, 2021: community-based cross-sectional study. Obstetr Gynecol Int. 2022;2022:1–11. 10.1155/2022/8086793 PMC9110235 35586393

[18] Kobiro DH , Delil R , Tamiru D , et al. Determinants of dietary diversity among pregnant women attending public health facilities in Hossana town, South Ethiopia. Research Square; 2019. DOI: 10.21203/rs.2.11374/v2.

[19] Hailu S , Woldemichael B. Dietary diversity and associated factors among pregnant women attending antenatal care at public health facilities in Bale Zone, Southeast Ethiopia. Nutr Diet Suppl. 2019;11:1.

[20] Jemal K , Awol M. Minimum dietary diversity score and associated factors among pregnant women at Alamata General Hospital, Raya Azebo Zone, Tigray Region, Ethiopia. J Nutr Metab. 2019;2019:1–6. 10.1155/2019/8314359 PMC6525861 31192011

[21] Saaka M , Oladele J , Larbi A , et al. Dietary diversity is not associated with the hematological status of pregnant women resident in rural areas of northern Ghana. J Nutr Metab. 2017;2017:1–10. 10.1155/2017/8497892 PMC5267082 28168052

[22] Murakami K , Miyake Y , Sasaki S , et al. Education, but not occupation or household income, is positively related to favorable dietary intake patterns in pregnant Japanese women: the Osaka Maternal and Child Health Study. Nutr Res. 2009;29(3):164–172. 19358930 10.1016/j.nutres.2009.02.002

[23] Hatløy A , Hallund J , Diarra MM , et al. Food variety, socioeconomic status, and nutritional status in urban and rural areas in Koutiala (Mali). Public Health Nutr. 2000;3(1):57–65. 10786724 10.1017/s1368980000000628

[24] Naveena N. Importance of mass media in communicating health messages: an analysis. IOSR J Human Soc Sci (IOSR-JHSS). 2015;20(2):36–41.

[25] Shahbaz P , Haq S , Khalid UB , et al. Gender-based implications of the COVID-19 pandemic on household diet diversity and nutritional security in Pakistan. Br Food J. 2022;124(3):951–967.

